# Liquid–liquid phase separation of the microtubule-binding repeats of the Alzheimer-related protein Tau

**DOI:** 10.1038/s41467-017-00480-0

**Published:** 2017-08-17

**Authors:** Susmitha Ambadipudi, Jacek Biernat, Dietmar Riedel, Eckhard Mandelkow, Markus Zweckstetter

**Affiliations:** 1 Deutsches Zentrum für Neurodegenerative Erkrankungen (DZNE), von-Siebold-Str. 3a, 37075 Göttingen, Germany; 2 Deutsches Zentrum für Neurodegenerative Erkrankungen (DZNE), Ludwig-Erhard-Allee 2, 53175 Bonn, Germany; 3 Max-Planck-Institut für Biophysikalische Chemie, Am Fassberg 11, 37077 Göttingen, Germany; 4 CAESAR Research Center, Bonn, and MPI for Metabolism Research, Hamburg Outstation, 22607 Hamburg, Germany; 5 Department of Neurology, University Medical Center Göttingen, University of Göttingen, Waldweg 33, 37073 Göttingen, Germany

## Abstract

The protein Tau aggregates into tangles in the brain of patients with Alzheimer’s disease. In solution, however, Tau is intrinsically disordered, highly soluble, and binds to microtubules. It is still unclear what initiates the conversion from an innocuous phase of high solubility and functionality to solid-like neurotoxic deposits. Here, we show that the microtubule-binding repeats of Tau, which are lysine-rich, undergo liquid–liquid phase separation in solution. Liquid–liquid demixing causes molecular crowding of amyloid-promoting elements of Tau and drives electrostatic coacervation. Furthermore, we demonstrate that three-repeat and four-repeat isoforms of Tau differ in their ability for demixing. Alternative splicing of Tau can thus regulate the formation of Tau-containing membrane-less compartments. In addition, phosphorylation of Tau repeats promotes liquid–liquid phase separation at cellular protein conditions. The combined data propose a mechanism in which liquid droplets formed by the positively charged microtubule-binding domain of Tau undergo coacervation with negatively charged molecules to promote amyloid formation.

## Abstract (web summary)

Tau forms aggregates in the brains of Alzheimer patients. Here, the authors identify conditions, where the microtubule-binding repeats of Tau undergo a phosphorylation-dependent liquid–liquid phase separation, leading to molecular crowding in the formed Tau liquid droplets and characterize them by NMR and other biophysical methods.

## Introduction

Alzheimer’s disease (AD) and several other neurodegenerative diseases are characterized by the misfolding and pathological accumulation of the microtubule-associated protein Tau^1–5^. The level of aggregation of Tau into neurofibrillary tangles (NFTs) correlates with the progressive destruction of nerve cells and the degree of cognitive decline in AD^6, 7^. Mutations in the Tau sequence modulate Tau’s ability to form tangles and cause frontotemporal dementia and parkinsonism linked to chromosome 17^8, 9^. Aggregated Tau is unable to bind to microtubules, which changes the dynamic instability of microtubules^10, 11^. Despite the pathological importance of misfolding and aggregation of Tau, the mechanisms underlying aberrant Tau aggregation and the pathways leading to tangle formation and neurotoxicity in AD have remained enigmatic.

Alternative splicing of exons 2, 3, and 10 of the Tau encoding *MAPT*-gen on chromosome 17 generates six different isoforms of Tau in the human central nervous system^12^. Tau isoforms differ in the number of N-terminal inserts and contain either three or four 31-residue to 32-residue-long imperfect repeat sequences (R1–R4). The repeat sequences are important for binding and assembly of microtubules and influence Tau’s ability to form tangles^13, 14^. In agreement with the importance of Tau repeats for pathogenic accumulation, the composition of tangles by 3R-Tau and 4R-Tau is connected to distinct clinical manifestations^1^. NFTs in AD contain a mixture of 4R-Tau and 3R-Tau^1^, while deposits in progressive supranuclear palsy predominantly contain 4R-Tau^15^, and Pick’s disease inclusions mostly have 3R-Tau^16^.

When Tau was purified as a microtubule-associated protein in 1975, it was recognized that it is highly soluble^17^. Because of the small number of hydrophobic residues and the low complexity of its amino acid sequence, Tau shows very little tendency to aggregate in vitro^18^. In addition, the protein does not fold into a stable structure^17^, but fluctuates between many different conformations^19^. This behavior is typical of intrinsically disordered proteins^20–22^. In order to undergo a liquid-to-solid phase transition and aggregate into tangles, the presence of polyanionic factors such as heparin and RNA or a specific yet unknown phosphorylation pattern are critical^23, 24^. NFTs isolated from the brains of patients with AD contain RNA and glycosaminoglycans^23^. In addition, proteases and endopeptidases liberate the repeat domain of Tau^25, 26^, which has higher aggregation propensity than the full-length protein^18^.

Pick’s disease and other frontotemporal dementias are associated with inclusions of both Tau and the RNA-binding protein fused in sarcoma (FUS)^8, 27^. Recently, it was shown that before FUS converts into insoluble deposits, it undergoes liquid–liquid phase separation (LLPS)^28–30^. Binding of FUS to RNA enhances the formation of liquid droplets. In addition, disease mutations in FUS leading to amyotrophic lateral sclerosis accelerate the transition from the liquid demixed state to an aberrant aggregated form^28, 29^. The identification of LLPS as an important step in FUS misfolding is in agreement with the increasing role of LLPS in biology and pathology^31^.

Eukaryotic cells have several membrane-less structures with liquid-like behavior such as P granules, nucleoli, and stress granules^29, 32–35^. They are often enriched in multivalent molecules^36^, including intrinsically disordered protein regions and proteins that contain multiple modular domains^37–39^. Indeed, several intrinsically disordered proteins and low-complexity regions in proteins were shown to drive LLPS^40^. Within membrane-less cellular bodies the concentration of proteins is higher than those of the crowded cytoplasm and nucleoplasm^39^. In addition, their physiochemical properties are distinct from the surrounding aqueous environment^41^, providing the means to spatially organize and biochemically regulate information.

Here, we show that the microtubule-binding repeats of Tau form liquid droplets in a phosphorylation-specific manner. Nuclear magnetic resonance (NMR) spectroscopy provides a residue-specific view into the structural properties of the phase-separated state and reveals crowding of amyloid-promoting elements upon liquid demixing. We further show that binding of negatively charged factors to Tau is not sufficient to trigger Tau amyloid formation, but requires coacervation into liquid droplets. In addition, three-repeat and four-repeat isoforms of Tau differ in their ability for LLPS, indicating that alternative splicing of pre-mRNA can influence the formation of Tau-containing membrane-less compartments in the reducing environment of a neuronal cell body.

## Results

### The propensity of Tau for granule formation

**Fig. 1:**
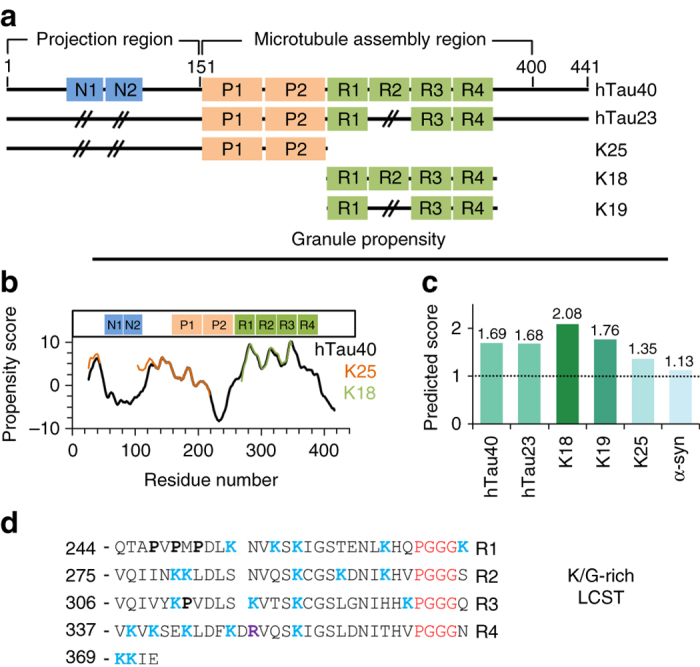
The Tau sequence encodes a strong propensity for lower-critical solution transition and granule formation. **a** Domain organization of the Alzheimer-related protein Tau. The longest Tau isoform (2N4R, htau40) in the human central nervous system contains four imperfect repeats (R1–R4) and two N-terminal inserts (N1, N2). htau23 (0N3R) is the shortest isoform and lacks the two N-terminal inserts as well as repeat R2. The Tau repeats form the core of NFTs^87^. The protein K25 contains only the N-terminal half of Tau, which is called the projection region. **b** Residue-specific propensity score for granule formation predicted by catGranule for full-length 4R-Tau (hTau40), the repeat domain of 4R-Tau (K18), and K25. **c** Total catGranule-scores of different Tau proteins and α-synuclein (α-syn). **d** Amino-acid sequence of repeat domain of Tau. It contains 19 lysine residues (highlighted in *blue*) and four PGGG motifs (*red*)

The amino acid sequence of human Tau contains only a small number of hydrophobic residues and is of low complexity^17^. To investigate if the primary structure of Tau encodes the ability for liquid–liquid demixing, we subjected the sequence of hTau40, the longest isoform in the human central nervous system, hTau23, which lacks the N-terminal inserts and repeat R2, as well as K18 (the repeat region of 4R-Tau; Fig. 1a) to the program catGranule^42^. catGranule predicts on the basis of structural disorder, nucleic acid-binding propensities, and sequence composition whether a protein is prone to granule formation. Figure 1b shows the catGranule-propensity score along Tau’s amino acid sequence. Propensity scores below zero were predicted for the two N-terminal inserts, part of the proline-rich region P2 and Tau’s C-terminal 40 residues. The largest values were located within the three imperfect repeats R2–R4 (Fig. 1b).

The overall granule propensity calculated with catGranule for hTau40 was 1.69 (Fig. 1c). Thus, the granule propensity of hTau40 is by 1.69 standard deviations larger than that predicted across the yeast proteome^42^. The 4R-region (K18) even reaches a granule propensity of more than 2.0, i.e., well above values of proteins that were experimentally shown to undergo liquid–liquid demixing. Notably, the protein α-synuclein, which aggregates into Lewy bodies in Parkinson’s disease^43, 44^, has a lower predicted propensity for granule formation when compared to Tau, but could still undergo liquid–liquid demixing according to the catGranule score (Fig. 1c).

### LLPS of the microtubule-binding domain of Tau

**Fig. 2:**
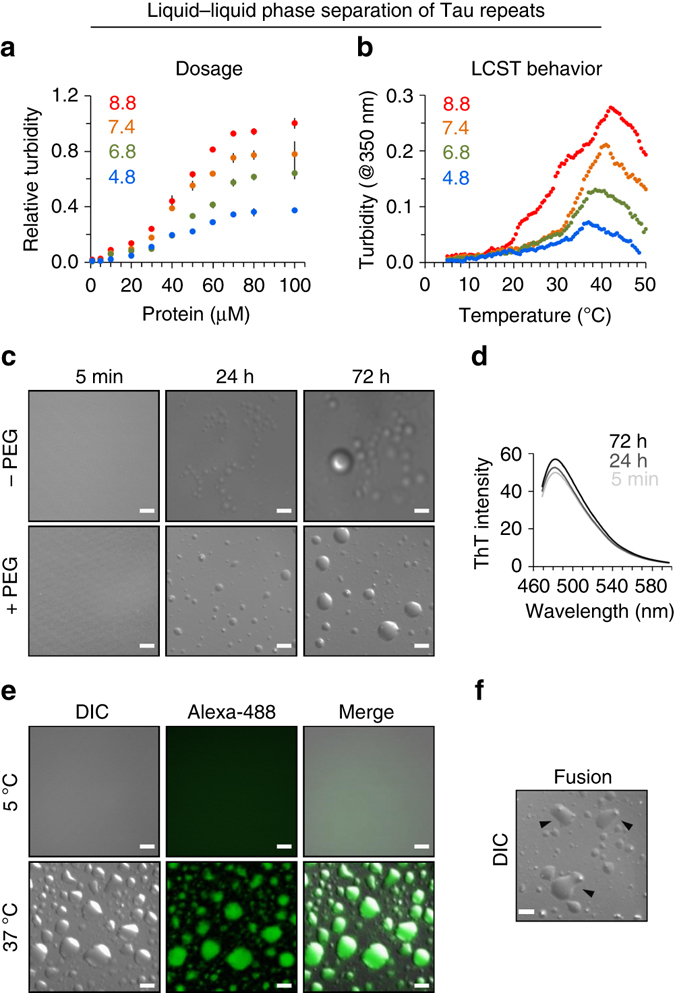
The microtubule-binding domain of Tau undergoes LLPS. **a** Influence of protein concentration and pH on turbidity of a K18 solution (50 mM sodium phosphate) at 37 °C. Turbidity values are reported as average absorbance at 350 nm from triplicate measurements for each sample. Normalization was done with respect to A(max) at pH 8.8, 100 μM K18 concentration. Errors were propogated as normalized standard error of mean (SEM). Increasing pH values are colored from *blue* to *red*. Note that all experiments were done in the presence of 0.5 mM TCEP, to avoid oxidation of Tau’s native cysteine residues. **b** K18 (100 μM in 50 mM sodium phosphate) undergoes liquid–liquid demixing above a critical temperature (~15 °C), consistent with a LCST. Increasing pH values are colored from *blue* to *red*. **c** Differential interference microscopy reveals the time-dependent formation of liquid droplets in a 100 μM solution of K18 (50 mM sodium phosphate, pH 8.8) at 37 °C, in both the absence (*top row*) and presence (*bottom*) of the molecular crowding agent PEG (7.5%). *Scale bars* correspond to 10 μm. **d** ThT fluorescence intensities for the samples imaged in **c** (no PEG) by DIC microscopy. Average intensities from three independent measurements are shown. **e** Fluorescence microscopy demonstrates the presence of K18 in liquid droplets formed at 37 °C (100 μM K18 in 50 mM sodium phosphate, pH 8.8). At 5 °C, K18 LLPS did not occur (*upper row*). Alexa-488-labeled protein was mixed with unlabeled protein in a molar ratio of 1:20. *Scale bars* correspond to 10 μm. **f** Fusion of K18 liquid droplets. Droplets, which were undergoing fusion when imaged, are marked by *black arrows*. The sample was identical to the one used in **c** (72 h; with PEG). *Scale bars* correspond to 10 μm

The computational analysis suggests that the Tau repeats, which are liberated from the full-length protein by proteases and endopeptidases^25, 26^, have a strong propensity for liquid demixing. We therefore subjected a solution containing the 4R-protein K18 to a wide range of temperatures, pH values, and protein concentrations (Fig. 2). This screen is important because protein-LLPS depends on the properties of the aqueous solution^45^. To exclude the influence of intramolecular and intermolecular cross-linking through Tau’s two native cysteine residues, C291 and C322^46^, all experiments were preformed in the presence of tris(2-carboxyethyl)phosphine (TCEP), mimicking the reducing environment inside neurons. We then used a standard assay to monitor liquid–liquid demixing of proteins, which is based on turbidity measurements at 350 nm. Indeed for pH values from 4.8 to 8.8 and a temperature of 37 °C, the K18 solution became more turbid with increasing protein concentration (Fig. 2a). Higher turbidity values were measured at more basic pH values approaching the protein’s pI of 9.8.

**Fig. 3:**
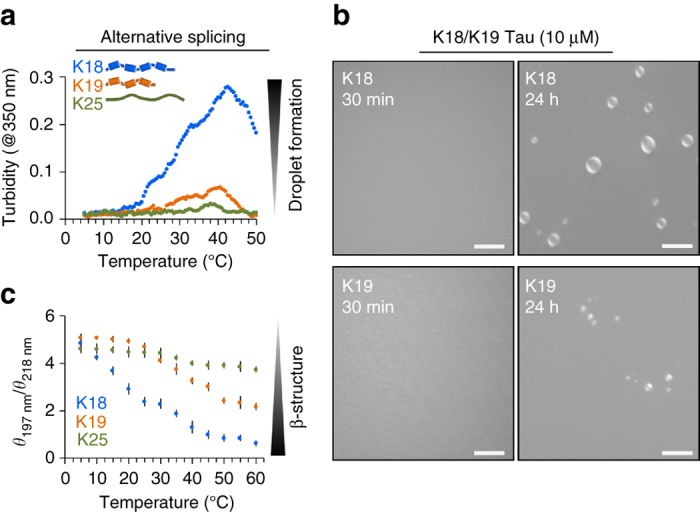
Alternative RNA splicing influences liquid demixing of the microtubule-binding domain of Tau. **a** Temperature-dependent changes in turbidity (at 350 nm) of solutions containing K19 (repeat region of 3R-Tau; *orange*) and K18 (repeat region of 4R-Tau; *blue*). Both samples contained 100 μM protein in 50 mM sodium phosphate, pH 8.8 with 0.5 mM TCEP. Because of the presence of TCEP in the samples, C291/C322 (K18) and C322 (K19) were not oxidized, i.e., no intermolecular or intramolecular disulfide bonds were formed, which would differentially influence aggregation of 3R-Tau and 4R-Tau^46^. The N-terminal half of Tau called projection region (K25; *green*; same sample conditions), which does not contain repeat sequences, did show at best very small changes in turbidity. **b** Liquid droplets observed for solutions of 10 μM K18 and 10 μM K19—both incubated at 37 °C—by DIC microscopy. *Scale bars* correspond to 10 μm. Buffer conditions were identical to **a**. Although DIC microscopy is not quantitative, multiple measurements on different samples consistently showed a larger number of droplets after 24 h of incubation at 37 °C in case of K18, i.e., the repeat region of 4R-Tau. No droplets were observed after 30 min. **c** Temperature-dependent changes in CD spectra of K18 (*blue*), K19 (*orange*), and K25 (*green*). Changes in β-structure were estimated from the ratio *θ*_197 nm_/*θ*_218 nm_. Smaller *θ*_197 nm_/*θ*_218 nm_ values are indicative of an increase in β-structure. *Error bars* represent SEM of three independent measurements (10 scan averages)

Changes in solution turbidity can arise from liquid–liquid demixing/LLPS, but also from formation of other types of aggregates. To support the presence of a liquid phase separated state of the repeat domain of Tau, we performed differential interference contrast (DIC) microscopy. Phase-contrast microscopy is particularly powerful for characterization of LLPS, because it reveals micrometer-sized structures, which are not visible with simple bright-field microscopes. Figure 2c shows DIC micrographs of a 100 μM solution of K18 at 37 °C, i.e., solution conditions that are optimal for K18 LLPS according to turbidity measurements (Fig. 2a), over a time period of 72 h. At the start of the imaging process, the solution is clear, indicating that K18 is present in solution as dispersed monomer. After 24 h, small droplets were observed. The number and diameter of droplets increased with time and after 72 h both large droplets with a diameter of approximately 15 μm and smaller (1 μm and below) droplets were observed. To mimic conditions of intracellular crowding, DIC micrographs were also recorded in the presence of 7.5% polyethylenglycol (PEG)^47^, supporting the time-dependent formation of K18 liquid droplets (Fig. 2c). The K18 liquid-demixed phase exhibited only little Thioflavin-T (ThT) fluorescence, indicating the absence of rigid cross-β-structure (Fig. 2d). The absence of amyloid-like structure is in agreement with the high solubility of K18, which requires an aggregation enhancer such as heparin to form amyloid fibrils at 37 °C, 100 μM protein concentration, on a time scale of 1–3 days^48^. Detailed inspection of DIC micrographs showed that the droplets were able to fuse (Fig. 2f), in agreement with their liquid-like nature. Liquid droplets were also observed by DIC microscopy for solutions containing lower protein concentrations, e.g., at 10 μM K18 (Fig. 3b).

To demonstrate the presence of K18 in the liquid droplets, we performed confocal microscopy of fluorescently labeled protein. To this end, we labeled K18 with the fluorescent dye Alexa-488. The fluorescently labeled protein was then mixed with unlabeled K18 in a molar ratio of 1:20, to reach a final concentration of 100 μM, pH 8.8. The solution was subsequently incubated for 12 h at both 5 and 37 °C and analyzed by microscopy. Only when incubated at 37 °C, but not at 5 °C, fluorescent droplets were observed (Fig. 2e). Taken together, the data show that the microtubule-binding domain of Tau undergoes LLPS at physiological conditions.

### Driving a lower critical solution transition (LCST)

Next, we fixed the protein concentration and varied the temperature from 5 to 50 °C (Fig. 2b). Below 15 °C we detected little solution turbidity. In addition, no evidence for liquid demixing was found at 5 °C by DIC microscopy (Fig. 2e). Thus, at high concentrations but low temperatures, K18 is present as dispersed monomer in solution. Above 15 °C, turbidity values rose, reached a maximum, and decreased at even higher temperatures. The maximum in turbidity was dependent on the pH of the solution and shifted from ~37 °C at pH 4.8 to ~42 °C at pH 8.8. Thus, the repeat region of Tau undergoes a so-called LCST^45^.

The dependence of LLPS on temperature is governed by the protein’s amino acid composition^45^. While a high content of arginine residues favours phase separation below a critical temperature, as observed for FUS, the presence of lysine residues has been associated with LCST^45^. In addition, P–X_*n*_–G motifs in intrinsically disordered peptides and proteins encode LCST behavior^45^. In agreement with these sequence determinants for phase separation at increasing temperature, K18 contains only a single arginine residue, but 19 lysine residues, as well as an evolutionary-conserved PGGG motif at the N-terminal end of each Tau repeat (Fig. 1d). In contrast to K18, domains of proteins, which were previously shown to undergo LLPS (e.g., FUS), are glutamine/asparagine-rich and undergo liquid demixing only at low temperature (below 20 °C)^28–30^.

### LLPS promotes reversible β-structure in Tau repeats

**Fig. 4:**
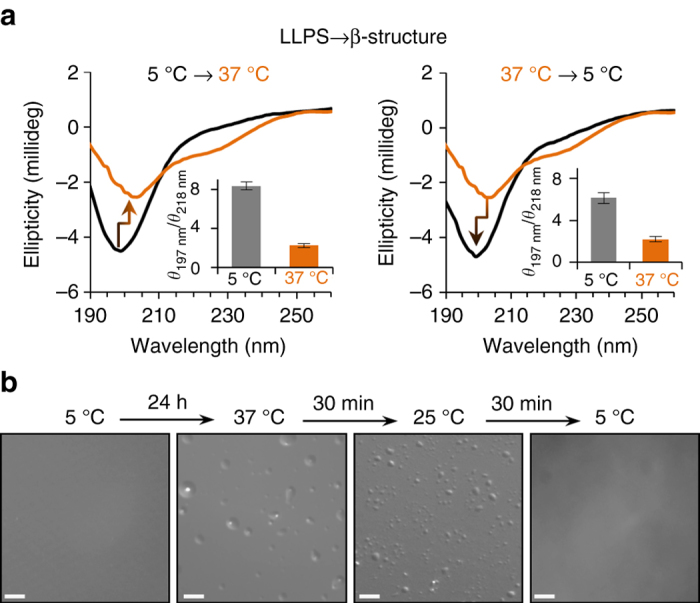
K18 phase separation is reversible. CD spectroscopy (**a**) and DIC microscopy (**b**) of K18 at 5 °C (dispersed monomer) and 37 °C (LLPS), and after return to 5 °C. *Scale bars* correspond to 10 μm. In **a**, *error bars* represent SEM of five measurements

To gain insight into structural properties of Tau repeats induced by aqueous phase separation, we performed circular di﻿chroism (CD) spectroscopy. In agreement with the intrinsically disordered nature of Tau^17^, CD spectra of K18, recorded at 5 °C, were characteristic for random coil behavior (Fig. 4a). Upon increasing the temperature to 37 °C, the minimum of the CD signal became less pronounced and shifted to longer wavelengths. Besides spectral changes at ~197 nm, the CD signal at 218 nm increased at 37 °C, indicative of increased β-structure. The structural changes were largely reversible after returning to 5 °C (Fig. 4a), in agreement with the disappearance of liquid droplets (Fig. 4b). Thus, the amount of β-structure in the Tau repeats is increased in solution conditions that promote LLPS. The increase in β-structure, however, is little when compared to K18 amyloid fibrils^49^, in agreement with very low ThT fluorescence in the liquid demixed state (Fig. 2d).

### Crowding of Tau amyloid hot spots upon liquid demixing

**Fig. 5:**
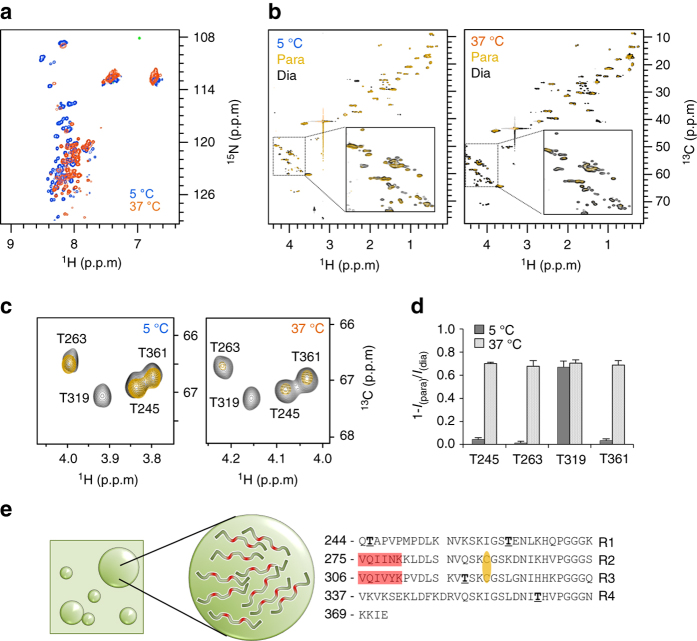
Liquid demixing causes molecular crowding of amyloid hot spots of Tau. **a** Two-dimensional ^1^H-^15^N HSQC spectra of the repeat domain of Tau (100 μM of K18 in 50 mM sodium phosphate, pH 8.8, and 0.5 mM TCEP) in the monomeric dispersed (*blue*; 5 °C) and liquid demixed (*red*; 37 °C) state. Due to increased solvent exchange, many ^1^H-^15^N cross-peaks of K18 are attenuated beyond detection at 37 °C^88^. In addition, an overall shift of the backbone resonances was observed, which is caused by the temperature dependence of NMR chemical shifts^52^. **b** Paramagnetic broadening in K18 (100 μM; sodium phosphate; pH 8.8), which was tagged with the nitroxide tag MTSL at the two native cysteine residues C291 and C322, was quantified in 2D ^1^H-^13^C HSQC spectra at 5 °C (*left*) and 37 °C (*right*). Paramagnetic and diamagnetic states are shown in *gold* and *black*, respectively. The Cα-Hα region of each spectrum is highlighted in the *insets*. **c** Selected region of the ^1^H-^13^C HSQC spectra shown in **b**, highlighting paramagnetic broadening of the four threonine residues in K18. **d** Quantification of paramagnetic broadening observed in ^1^H-^13^C HSQC spectra shown in **b** and **c**. Upon liquid demixing at 37 °C (100 μM protein concentration; also see Fig. 2), ^1^H-^13^C cross-peaks of T245, T263, T319, and T361 were strongly attenuated (1-*I*_para_/*I*_dia_). In contrast, at 5 °C where K18 is present as dispersed monomer (Fig. 2), only T319, which is in direct proximity to the MTSL-carrying C322, was broadened. *Error bars* are calculated based on the signal-to-noise in the NMR spectrum of diamagnetic K18. **e** Schematic representation of molecular crowding of Tau’s aggregation-prone hexapeptides (*red*) in the interior of liquid droplets. The sequence location of the two hexapeptides and Tau’s native cysteine residues (*yellow*; attachment sites of MTSL) is shown on the *right*. Threonine residues are highlighted in *bold*

A critical aspect of liquid–liquid demixing is that the resulting membrane-less compartments have distinct physiocochemical properties^50^. Quantitative studies of liquid droplets, however, are complicated by their liquid nature and the rapid exchange with the surrounding environment. To dissect the consequences of LLPS on the molecular properties of the repeat domain of Tau, we used NMR spectroscopy. NMR spectroscopy not only provides residue-resolution, but is also able to characterize molecules that exchange between multiple states^51^. A two-dimensional ^1^H-^15^N correlation spectrum, recorded for the repeat domain of Tau at 5 °C, 100 μM protein concentration, had small signal dispersion (Fig. 5a), in agreement with a dispersed monomeric protein (Fig. 2e). At 37 °C, the temperature at which DIC microscopy demonstrated the presence of phase separation (Fig. 2c, e), the small signal dispersion remained but an overall upfield shift of the backbone amide proton resonances was observed (Fig. 5a). This shift is caused by the temperature dependence of NMR chemical shifts^52^ and therefore does not necessarily report on changes in chemical environment. In addition, peak intensity losses at 37 °C are due to more rapid solvent exchange at higher temperatures. Notably, no additional NMR signals were found at 37 °C, which would report on alternative states/conformations of the repeat domain of Tau in the liquid demixed state. The NMR data suggest that the protein stays largely disordered within liquid droplets, in agreement with the overall low content of regular secondary structure observed by CD spectroscopy (Fig. 4a). However, because the NMR data were obtained on a phase-separated mixture of protein-dense and protein-depleted states, the quality of the ^1^H-^15^N spectrum was decreasing at higher temperature, and CD spectroscopy is not sensitive enough for local structural changes in an otherwise disordered protein, specific structural features such as turns or salt bridges in the condensed state cannot be detected.

Because liquid droplets are not bounded by membranes, molecules rapidly exchange in and out. If this exchange is fast on the NMR time scale, NMR spectra in liquid demixed phases will report on the average properties of the protein when being present in the interior of liquid droplets and as dispersed monomer in the surrounding environment. The observation of largely unchanged chemical shifts of K18 (Fig. 5a) in a mixture of protein-dense and protein-depleted states is therefore either due to (i) largely identical chemical environments for K18 residues in the interior of liquid droplets and as dispersed monomer (in agreement with CD spectroscopy; Fig. 4a) or (ii) the fast exchange of K18 molecules from the interior of the liquid droplets into the surrounding environment (in agreement with the basic properties of liquid droplets) or (iii) a combination of the two. Because of the fast exchange in and out of liquid droplets, the NMR properties of K18 in conditions of LLPS might be similar to those of ligands that weakly bind to receptors^51^. To probe this hypothesis, we attached the paramagnetic nitroxide tag (1-oxy-2,2,5,5-tetramethyl-d-pyrroline-3-methyl)-methanethiosulfonate (MTSL) to the two native cysteines, C291 and C322, of K18 (Fig. 5b, e). Paramagnetic centers in proteins strongly increase relaxation rates of nearby nuclei^53^ and thereby provide highly sensitive probes for both intramolecular and intermolecular interactions^19, 54^. The paramagnetic effects induced by the two MTSL-tags in K18 (protein concentration: 100 μM) were quantified through ^1^H-^13^C correlation spectra (Fig. 5b). ^1^H-^13^C correlation spectra are advantageous compared to ^1^H-^15^N correlation spectra, because they are less affected by solvent exchange, in particular at 37 °C. The NMR analysis showed that at 5 °C, when the protein is present primarily as dispersed monomer (Fig. 2e), only residues in the immediate vicinity of the two cysteine residues such as T319 were broadened (Fig. 5b–e). In contrast, most K18 residues felt the presence of the paramagnetic center in the liquid–liquid demixed state at 37 °C (Figs. 2c, 5b). Indeed, all four threonine residues, which were well separated in ^1^H-^13^C correlation spectra (Fig. 5c), were strongly broadened (Fig. 5c, d).

In the monomeric dispersed state, only weak intramolecular and no intermolecular interactions are present in both full-length Tau and K18^19, 49^. The observation of severe resonance broadening in K18 at 37 °C, 100 μM protein concentration (Fig. 5c) thus demonstrates that Tau’s four repeats tightly interact with each other in the conditions, in which phase separation in solution occurs (Fig. 2c). The contacts can be within a single protein, as well as between different molecules. Importantly, the observed molecular contacts provide direct evidence for crowding of the repeat domain of Tau and its aggregation-prone hexapeptides. In contrast, we did not observe such strong molecular interactions in K18—even in the presence of the aggregation enhancer polyglutamic acid—when the temperature was kept at 5 °C^49^, i.e., below the critical temperature for LLPS of K18 (Fig. 2). The data demonstrate that LLPS of Tau repeats results in a tight molecular mesh of amyloid-promoting elements (Fig. 5e).

### LLPS promotes Tau fibrillization in the presence of heparin

**Fig. 6:**
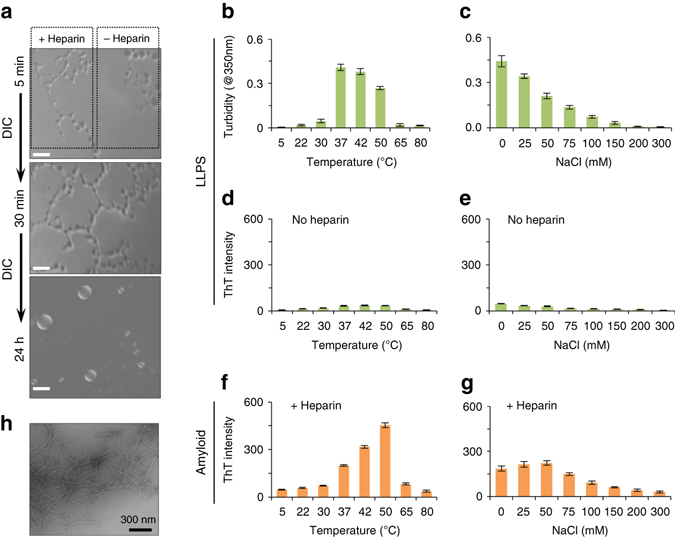
LLPS of the repeat domain of Tau promotes fibrillization in presence of the polyanion heparin. **a** Addition of heparin to K18 droplets (100 μM of K18 in 50 mM sodium phosphate; 0.5 mM TCEP; pH 8.8; 37 °C; K18:heparin molar ratio of 4:1) results in formation of a phase-separated mesh. DIC micrographs show that the region, to which heparin was added (shown along *dotted lines*; +heparin), changes first. After 24 h, the mesh has dissolved and larger liquid droplets were observed. *Scale bars* correspond to 10 μm. **b**, **d** Influence of temperature on turbidity (**b**) and ThT fluorescence (**d**) of a K18 solution (100 μM K18 in 50 mM sodium phosphate, 0.5 mM TCEP, pH 8.8) in the absence of heparin. Prior to turbidity measurements, solutions were incubated at the specified temperature for 6 h. Error bars in **b**–**g** represent SEM of three independent measurements. **c**, **e** Ionic strength dependence of turbidity (**c**) and ThT fluorescence (**e**) of heparin-free K18 solutions (100 μM K18 in sodium phosphate, 0.5 mM TCEP, pH 8.8). Prior to turbidity measurements, solutions were incubated at 37 °C for 24 h. **f** Temperature-dependence of amyloid formation of K18 (100 μM K18 in 50 mM sodium phosphate, pH 8.8) in the presence of heparin (K18:heparin molar ratio 4:1). Samples were incubated for 3 days at different temperatures (*x*-axis). At the end of the incubation period, amyloid formation was probed by ThT fluorescence measurements (*y*-axis). Because of the presence of 0.5 mM TCEP, K18 cysteine residues were not oxidized during the experiment. **g** Ionic strength dependence of K18 fibrillization in the presence of heparin (K18:heparin ratio 4:1; sodium phosphate, 0.5 mM TCEP, pH 8.8). Separate samples with increasing NaCl concentrations (*x*-axis) were prepared and incubated for 3 days at 37 °C. **h** Aggregation of K18 into amyloid fibrils in the presence of heparin after 3 days of incubation at 37 °C imaged by electron microscopy

Polyanions such as glycosaminoglycans and ribonucleic acids promote the aggregation of Tau and are found in NFTs isolated from the brains of patients with AD^23, 24^. A well-studied polyanion is heparin^48^, which binds to the repeat domain of Tau^55^. To investigate if LLPS is related to formation of insoluble protein deposits, we added heparin to a selected region of a K18 sample containing preformed liquid droplets. Contrast microscopy showed that 5 min after addition of heparin liquid droplets were connected by tube-like structures (Fig. 6a, *upper panel*). The phase-separated mesh was only observed in the region of the specimen where heparin had been added, while separated droplets remained in a distant region (Fig. 6a, *upper panel*). Twenty-five minutes later, the mesh-like network had spread over the whole sample (Fig. 6a, *middle panel*). After 1 day of incubation in the presence of heparin, larger liquid droplets were observed by contrast microscopy (Fig. 6a, *lower panel*). After 2 more days of incubation, abundant amyloid fibrils of K18 were observed by electron microscopy (Fig. 6h).

In order to provide further support for the importance of LLPS for polyanion-induced Tau aggregation, we compared in a very systematic manner the influence of temperature and ionic strength on heparin-free LLPS (Fig. 6b–e) with heparin-induced K18 fibrillization (Fig. 6f, g). Liquid demixing in the absence of heparin was strongly dependent on temperature (Fig. 6b). Little turbidity was observed at low temperature, in agreement with a LCST (Fig. 2b). The absence of LLPS at 5 °C was also supported by DIC microscopy (Fig. 2e). In addition, no evidence for liquid–liquid demixing was found at very high temperatures (65 °C and above; Fig. 6b), demonstrating that the absence of LLPS at 5 °C is not related to a very slow kinetic process. The detailed temperature scan shown in Fig. 6b revealed a maximum in solution turbidity at ~42 °C, suggesting that close to physiological temperatures are optimal for liquid demixing of K18. At all tested temperatures, very little ThT fluorescence was detected (Fig. 6d), demonstrating the absence of amyloid fibrils in heparin-free conditions.

**Fig. 7:**
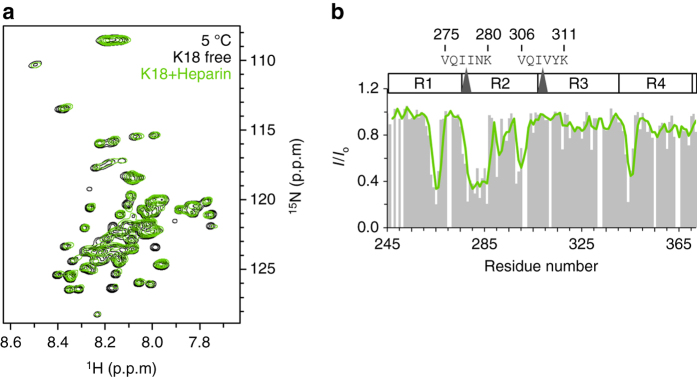
Heparin binds to the dispersed K18 monomer at 5 °C. **a** 2D ^1^H-^15^N HSQC spectrum of K18 (100 μM) in the absence (*black*) and presence (*green*) of heparin (K18:heparin molar ratio of 4:1) recorded at 5 °C. **b** Residue-specific changes in NMR signal intensity demonstrate binding of heparin to K18; however, no fibrils were formed despite incubation for 3 days (Fig. 6f). Signal intensities in the absence (*I*_0_) and presence of heparin (*I*) were taken from **a**. The *green line* shows the 3-residue average of *I*/*I*_0_. Aggregation prone hexapeptide sequences in R2 and R3 are highlighted

We then incubated K18 solutions with heparin for 3 days at different temperatures. At the end of the incubation period, the presence of fibrils was probed using the amyloid-specific dye ThT. With increasing incubation temperature the ThT intensity of the K18/heparin solution rose (Fig. 6f). Electron microscopy confirmed the presence of amyloid fibrils (Fig. 6h). Although ThT intensity is not a quantitative measure for protein fibrillization, the data suggest that K18 fibrillization in the presence of a K18:heparin molar ratio of 4:1 is most efficient at ~50 °C (Fig. 6f), in agreement with previous results^56^. When the temperature was increased beyond 50 °C, however, very little ThT fluorescence was detected. Thus, despite the presence of heparin, the repeat domain of Tau was not able to aggregate into amyloid fibrils at high temperatures. The data show that heparin-free LLPS and heparin-induced fibrillization of K18 show a similar temperature dependence: Only at intermediate temperatures close to 37 °C, but not a very low (<15 °C) or very high (>65 °C) temperatures K18 undergoes LLPS in the absence of heparin. Addition of heparin to K18 at temperatures, where no liquid demixing occurs, did not result in K18 fibrillization (Fig. 6f). Notably, the inability of K18 to form amyloid fibrils in the presence of heparin at 5 °C is not due to an impaired K18/heparin interaction, because NMR spectroscopy demonstrated that heparin still binds to K18 at 5 °C (Fig. 7).

The inability of K18 to aggregate into amyloid fibrils at very low (<15 °C) or very high (>65 °C) temperatures in the presence of one of its most potent aggregation enhancer heparin might be caused by temperature-dependent changes in hydrophobicity or entropy. To further tighten the connection between Tau LLPS and amyloid formation, we therefore systematically varied a second physiochemical parameter, ionic strength. Figure 6c shows that with increasing NaCl concentration the turbidity of a 100 μM solution of K18, pH 7.4, 37 °C, decreased. The data suggest that at a NaCl concentration of 200 mM, the protein was only present as dispersed monomer. We then assayed the influence of the NaCl concentration on heparin-induced K18 fibrillization. Similar to heparin-free LLPS, heparin-induced K18 fibrillization showed a strong dependence on ionic strength: at NaCl concentrations of 200 mM and above no amyloid fibrils were formed (Fig. 6g). The data indicate that electrostatic interactions are important for both heparin-free LLPS and heparin-induced fibrillization.

The combined data show that heparin-free LLPS and heparin-induced fibrillization depend in a similar way on temperature and ionic strength of K18 solutions. In addition, heparin-induced fibrillization of K18 is more efficient at basic pH values^56^, just as heparin-free LLPS (Fig. 2a). Thus, heparin-free LLPS and heparin-induced fibrillization of K18 have similar dependences on three distinct physiochemical solution properties: temperature, ionic strength, and pH. In addition, DIC microscopy revealed immediate changes in the liquid demixed state upon addition of heparin (Fig. 6a), which resulted in amyloid fibril formation upon continued incubation (Fig. 6h). Taken together, the data indicate that amyloid formation does not occur—in reducing conditions and in the presence of the aggregation enhancer—when the repeat domain of Tau is present as dispersed monomer. The data suggest a model, in which liquid demixing results in increased concentrations of the repeat domain in liquid droplets. Because the repeat domain is highly positively charged, polyanions are recruited to K18 liquid droplets, thereby increasing the local concentration of Tau/polyanion-complexes and promoting amyloid formation.

### Valency of homotypic Tau interactions

Due to alternative RNA splicing, Tau occurs in six different isoforms in the human central nervous system^12, 57^. The isoforms differ by the number of inserts in the N-terminal half and the number of repeats in the C-terminal half of the protein. We therefore asked if the number of repeats influences the ability for Tau LLPS. To this end, we prepared the protein K19. K19 comprises the repeat region of 3R-Tau (Fig. 1a). With increasing temperature, a rise in the turbidity at 350 nm of a K19 solution was observed (Fig. 3a), indicating that K19 undergoes aqueous phase separation. Observed turbidity values, however, were lower than for K18 over a wide temperature range. In agreement with a lower propensity of K19 (repeat region of 3R-Tau) for LLPS, a smaller number of liquid droplets were observed by contrast microscopy (Fig. 3b). Even lower turbidity values were detected for K25 (Fig. 3a), the Tau fragment, which comprises the projection domain but no repeats (Fig. 1a). In agreement with turbidity values and DIC microscopy, a smaller amount of β-structure was stabilized in K19 than K18 upon LLPS (Fig. 3c). The data suggest that each imperfect repeat in Tau can be regarded as a single interaction motif, with the number of Tau repeats governing the multivalency of homotypic interactions in liquid demixing.

### MARK2 phosphorylation promotes K18 LLPS

**Fig. 8:**
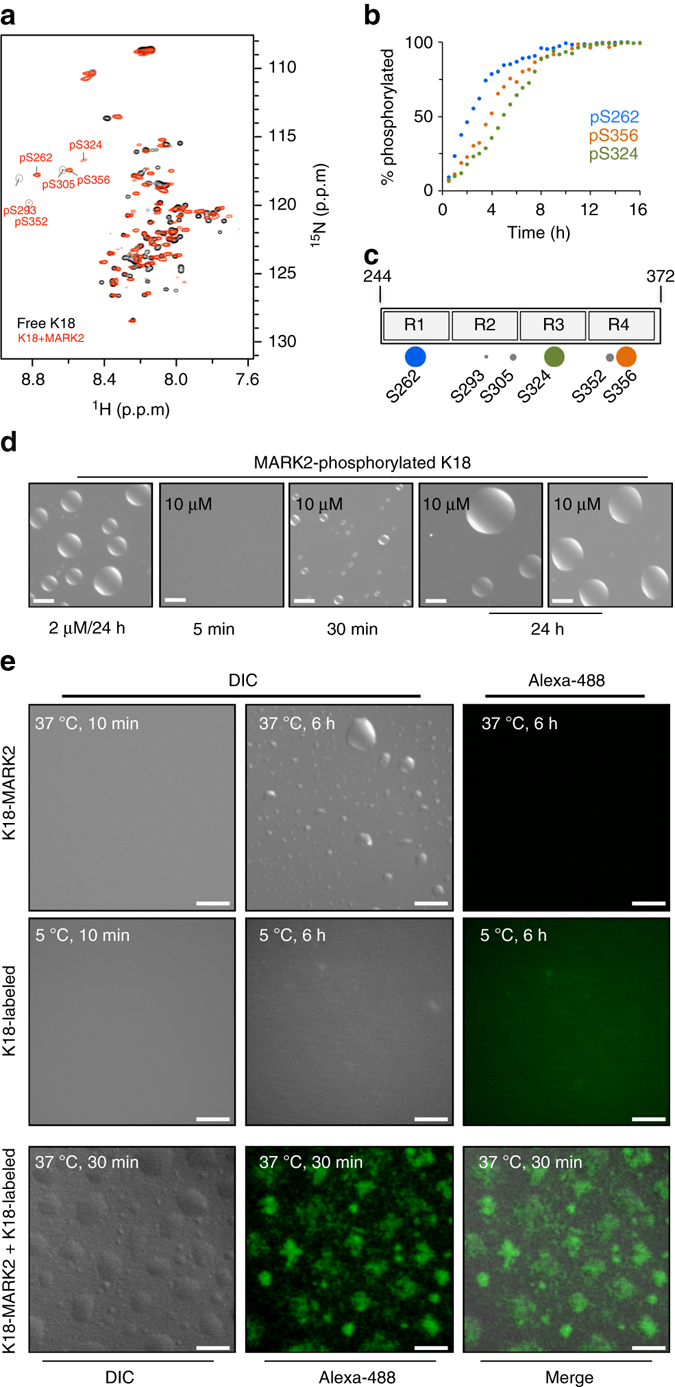
MARK2-phosphorylation lowers the critical concentration of K18 LLPS. **a** 2D ^1^H-^15^N HSQC spectrum of K18 (100 μM protein in 50 mM sodium phosphate, pH 6.8) prior to (*black*) and after (*red*) phosphorylation by MARK2. NMR experiments were recorded at 5 °C. Cross-peaks of phosphorylated serine residues are labeled. **b** Residue-specific kinetics of phosphorylation of K18 by MARK2. Errors in “% phosphorylated” were calculated on the basis of the signal-to-noise ratio in the spectra and were below 3%. Because S293, S305, and S352 were only phosphorylated by about 10–20%, reliable analysis of their phosphorylation kinetics was not possible. For details on in vitro phosphorylation of K18, please see the experimental section. **c** Schematic representation of the serine residues of K18 that were phosphorylated in vitro by MARK2. S262, S324, and S356 were 100% phosphorylated (*large dots*), in agreement with previous results^79^. **d** MARK2-phosphorylated K18 (50 mM sodium phosphate, pH 8.8, 0.5 mM TCEP) undergoes LLPS when incubated at a concentration of 2 μM at 37 °C (*left panel*), i.e., the concentration at which Tau is estimated to be present in neurons^60^. Increasing the concentration of MARK2-phosphorylated K18 further enhanced LLPS. For example, after 24 h of incubation large droplets were present (*right panel*). *Scale bars* correspond to 10 μm. Because DIC micrographs are not quantitative, the most representative images of a large number of recorded micrographs are shown. **e** Non-phosphorylated K18 is located to liquid droplets, which are primarily formed by MARK2-phosphorylated K18. Non-phosphorylated K18 was labeled with Alexa-488, MARK2-phosphorylated K18 did not carry a fluorescent tag

Phosphorylation of Tau precedes formation of insoluble Tau deposits^58^. Because microtubule-associated protein/microtubule affinity-regulating kinases (MARKs) phosphorylate serine residues in the repeat domain of Tau, MARK kinases influence both Tau aggregation and microtubule binding^59^. We therefore asked if phosphorylation by MARK2 modulates the ability of the repeat domain of Tau to undergo LLPS. To this end, we phosphorylated K18 by MARK2 (Fig. 8a). This resulted in complete phosphorylation of the serine residues S262, S324, and S356 (Fig. 8b). In addition, S293, S305, and S352 were phosphorylated by about 10–20% (Fig. 8a, c).

Initially, we again started with a low-protein concentration, at which non-phosphorylated K18 did not show any sign of phase separation by either DIC microscopy or turbidity measurements (Fig. 2). Strikingly, even at 2 μM, MARK2-phosphorylated K18 readily formed liquid droplets after incubation for 1 day at 37 °C (Fig. 8d, *left panel*). Because 2 μM is the estimated concentration of Tau in neurons^60^, the data demonstrate that MARK2-phosphorylated K18 undergoes LLPS at physiological protein concentrations.

When the concentration was raised to 10 μM, liquid droplet formation of MARK2-phosphorylated K18 was further promoted. After 30 min of incubation at 37 °C, micrometer-sized droplets were already observed (Fig. 8d). Continued incubation of MARK2-phosphorylated K18 at 37 °C further increased the diameter of droplets, now reaching up to 20 μm (Fig. 8d). Taken together, the data demonstrate that MARK2-phosphorylation promotes K18 LLPS.

We then asked whether non-phosphorylated K18 is recruited to liquid droplets formed by MARK2-phosphorylated K18. To this end, we labeled the non-phosphorylated protein with Alexa-488, while the MARK2-phosphorylated K18 was not fluorescently labeled. In agreement with previous results, MARK2-phosphorylated K18 formed micrometer-sized liquid droplets at 37 °C in a time-dependent manner (Fig. 8e). Because MARK2-phosphorylated K18 was not labeled with a fluorescent dye, the droplets did not show any fluorescence (Fig. 8e, *top panel*). As a further control, the Alexa-488 labeled non-phosphorylated protein did not form droplets over a time period of 6 h when incubated at 5 °C (Fig. 8e, *middle panel*). To test recruitment of non-phosphorylated K18 to liquid droplets of MARK2-phosphorylated K18, we prepared a fresh sample containing a 4:1 mixture of MARK2-phosphorylated K18 and non-phosphorylated Alexa-488-labeled K18, and incubated the sample at 37 °C for 30 min. DIC microscopy showed that droplets had formed (Fig. 8e, *lower panel*). Notably, the droplets were fluorescent suggesting that non-phosphorylated K18 was incorporated into liquid droplets (Fig. 8e, *lower panel*), which are primarily formed by MARK2-phosphorylated K18 (Fig. 8d).

## Discussion

**Fig. 9:**
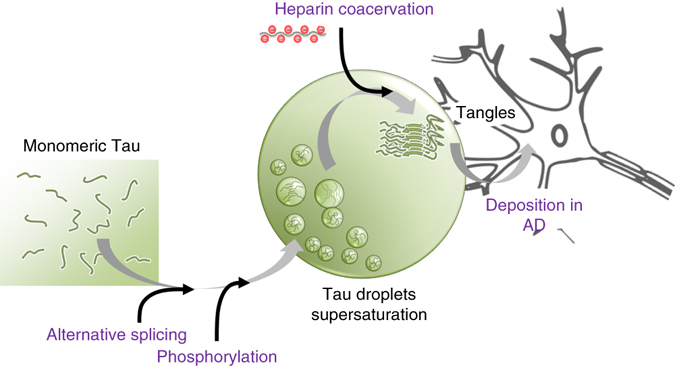
Isoform-specific and phosphorylation-dependent LLPS of the microtubule-binding domain of Tau is important for amyloid formation. Schematic representation illustrating liquid–liquid demixing of Tau repeats as an important step in the cascade of aberrant Tau misfolding from monomeric dispersed protein in solution to insoluble tangles. Critical events in this process are alternative splicing, which determines the number of Tau repeats, and MARK2 phosphorylation that lowers the critical concentration of LLPS. In the interior of liquid droplets a supersaturated state is present, which recruits polyanions through electrostatic coacervation, thereby promoting amyloid formation under the reducing environment of a cell

Pathological inclusions of the microtubule-associated protein Tau are present in several neurodegenerative diseases, collectively referred to as tauopathies^1–3^. Because the appearance of insoluble Tau deposits correlates with loss of neurons in patients with AD, Tau inclusions are believed to be important diagnostic markers and targets for therapeutic intervention^61, 62^. The pathological importance of Tau aggregation stands in strong contrast to our knowledge about the underlying mechanisms and pathways that lead from the innocuous phase of high solubility to aberrant disease-relevant Tau states in neurodegeneration. Here, we showed that the microtubule-binding repeats of Tau undergo LLPS in reducing condition at physiological temperature (Fig. 9). The droplets formed by the microtubule-binding domain of Tau are liquid-like (Fig. 2f) and do not contain amyloid fibrils (Fig. 2d), in agreement with the very high solubility of the repeat domain of Tau in reducing conditions^48^. Because of their large size (up to several micrometers), their spherical/circular shape (Fig. 2c, e), their reversibility (Fig. 4), and their molecular properties (Fig. 5), K18 liquid droplets are distinct from soluble aggregates of Tau including soluble oligomers. In the absence of phosphorylation, rather high protein concentrations (>~10 μM; Fig. 2b) were required to observe phase separation. Phosphorylation by the kinase MARK2, however, lowered the critical concentration for phase separation, resulting in liquid demixing already at a concentration of 2 μM (Fig. 8d), the estimated concentration of Tau in neurons^60^. Importantly, droplets formed by the microtubule-binding domain of Tau vary in size (Fig. 2), suggesting that also in the confined environment of a neuronal cell body, liquid droplets are feasible. Through the process of liquid–liquid demixing, the local concentration of the Tau repeat domain is strongly increased, which causes molecular crowding of amyloid-promoting Tau elements (Figs. 5, 9). In addition, liquid demixing establishes a Tau reaction chamber with a high positive charge density (the pI of the repeat domain of Tau is 9.8), which can recruit polyanionic aggregation enhancers such as heparin. The microtubule-binding domain of Tau is lysine-rich, while the domains of prior examples driving protein de-mixing (e.g., FUS, hnRNPA2B1) are glutamine/asparagine-rich. Thus, Tau de-mixing suggests new/different biochemical principles that can drive LLPS.

Supersaturation of proteins, proteins whose cellular concentration is high relative to their solubility, is an important mechanism for aberrant aggregation^63^. In contrast, the protein Tau is well below supersaturation according to known estimated concentrations of Tau in neurons (~2 μM^60^) and the protein’s very low intrinsic fibrillization propensity^18^. This analysis, however, does not include variations in concentration due to compartmentalization. We showed that liquid droplets formed by the Tau repeat region concentrate the most aggregation-prone Tau residues, the two hexapeptides ^275^VQIINK^280^ and ^306^VQIVYK^311^ at the beginning of repeats R2 and R3. Compartment-specific concentration results in the formation of a tight network of molecular interactions (Fig. 5). Within this protein-dense mesh, Tau’s hexapeptides are brought into spatial proximity. Liquid droplets formed by the repeat domain of Tau thus represent a supersaturated, metastable state of the protein that is distinct from the kinetically stable, monomeric innocuous Tau.

Protein inclusions such as NFTs in AD are solid-like. Within NFTs, Tau and its more aggregation-prone fragments are locked into cross-β-structure, resulting in long-term storage and inactivation^64^. In contrast, a characteristic property of liquid-like compartments, which are not bounded by a membrane, is the rapid exchange of molecules between the interior of these compartments and the surrounding cytoplasm^39, 65^. The observation of strong paramagnetic broadening of NMR signals, which however do not have unique chemical shifts but those of the monomeric dispersed protein (Fig. 5), indicates that the exchange between the interior of liquid droplets and the surrounding environment is fast on the NMR time scale. Indeed, the NMR properties of K18 in conditions of LLPS are radically different, when compared to oligomeric/higher order aggregates of K18. Over the many years that we have studied the Tau protein and K18, oligomer preparations always resulted in strong loss of signal intensities in the Cα-Hα region of ^1^H-^13^C heteronuclear single-quantum coherence (HSQC) spectra even in the absence of any paramagnetic tag. This NMR signal decrease in K18/Tau oligomers is due the slow (on the NMR time scale) exchange between the oligomeric/aggregated state and the monomeric protein, precluding up to this very day a more detailed structural analysis of Tau oligomers by NMR spectroscopy. Similar observations have been made for soluble oligomers of the Parkinson-related protein α-synuclein^66^. The slow exchange of K18/Tau molecules between the monomeric state and the oligomeric form is in strong contrast to our finding for K18 in liquid droplets, where signal broadening is only observed in the paramagnetic state, but not the diamagnetic protein (Fig. 5b), in agreement with the liquid-like nature of droplets formed by the microtubule-binding domain of Tau. At the same time, it cannot be excluded that for example a small fraction of soluble oligomers, which are not visible to NMR, might contribute to changes in the CD signal observed at 37 °C (Fig. 4a).

A variety of molecules have been demonstrated to promote fibrillization of Tau. Particularly efficient are polyanionic factors such as heparin and ribonucleic acids^23, 24^. Probing a large number of physicochemical conditions in a systematic manner, we showed that conditions, in which K18 undergoes LLPS, promote amyloid formation of the protein upon addition of heparin (Fig. 6). For example, in a solution of 100 μM K18 liquid droplets were formed when incubated at 37 °C in reducing conditions for 24 h (Fig. 2c, e). When then heparin is added in the optimal molar ratio of 4:1 (K18:heparin)^48^, amyloid fibrils are formed (Fig. 6a, f, g). In contrast, K18 did not form amyloid fibrils in conditions, in which we did not observe liquid demixing. At 5 °C, for example, the microtubule-binding domain of Tau did not phase separate according to DIC and fluorescence microscopy (Fig. 2e), in agreement with the nature of a LCST^45^. Although NMR spectroscopy demonstrated that heparin binds to K18 in this condition (i.e., at 5 °C; Fig. 7), the interaction was not able to convert the protein into amyloid fibrils (Fig. 6f). The data suggest that—in reducing conditions and at physiological temperature—LLPS is necessary to increase the local concentration of Tau repeats and recruit heparin, in order for the protein to misfold into amyloid fibrils. K18-heparin coacervation changes the phase properties of the solution, resulting in a phase-separated mesh (Fig. 6a) and aggregation into amyloid fibrils (Fig. 6h). A coacervation mechanism of Tau pathogenicity is in line with the concentration of RNA in liquid droplets formed by cationic peptides and proteins^67^.

In vivo additional factors, such as molecular chaperones^68, 69^, are likely to influence liquid–liquid demixing of the Tau repeats. In addition, membrane-less structures with liquid-like behavior such as P granules, nucleoli, and stress granules contain not a single, but several different proteins, with some of them binding RNA and potentially undergoing liquid–liquid demixing^29, 32–35^. Thus, heterotypic multivalent interactions will further influence the presence of Tau in liquid-like compartments. Consistent with this hypothesis, the Tau protein is present as speckles in the nuclei of SH-SY5Y human neuroblastoma cells^70^ and together with other proteins in stress granules of patients with AD^37, 71, 72^. Further studies will have to clarify how these different proteins work together to form Tau-containing membrane-less compartments. Notably, laser capture microdissection found 72 different proteins in NFTs^73^, indicating that protein co-aggregation is important for aberrant accumulation of Tau.

In the brains of patients with AD, the protein Tau is hyperphosphorylated^74–76^. Tau hyperphosphorylation precedes tangle formation and at least 39 phosphorylation sites have been identified in NFTs^77^. MARK kinases efficiently phosphorylate serine residues in the repeat domain of Tau^78, 79^ and inhibition of MARK kinases abrogates Aβ-induced cellular toxicity^80^. Contrast microscopy showed that phosphorylation by MARK2 changes the kinetics and dimensions of liquid droplets and promotes LLPS at lower protein concentrations (Fig. 8). In agreement with the importance of post-translational modifications for LLPS^67^, phosphorylation of the soluble tail of nephrin modulates liquid droplet formation of the N-WASP/NCK/nephrin system^36^. The influence of MARK-phosphorylation on liquid–liquid demixing of the Tau repeat domain suggests that recruitment of Tau into liquid-like cellular bodies can be controlled by kinases.

Alternative splicing of pre-mRNA generates multiple isoforms of Tau, which contain either three (3R) or four (4R) Tau repeats^12^. 3R-Tau and 4R-Tau differ in their ability to assemble microtubules and have different aggregation propensities^18, 81^. Consistent with the pathological importance of alternative splicing for Tau-related neurodegeneration, Tau inclusions in tauopathies with distinct clinical manifestations contain 3R-Tau and 4R-Tau at different molar ratios^1, 12^. Our study shows that the number of Tau repeats influences aqueous phase separation. In the absence of cysteine oxidation, K19 (3R-Tau) has a lower propensity for LLPS than K18 (4R-Tau) and a repeat-less Tau fragment (K25) did not undergo LLPS (Fig. 3). In agreement with attenuated phase separation, K19 contained less β-structure and repeat-less Tau (K25) experienced no time-dependent changes in structure. Moreover, aggregation of Tau into amyloid fibrils requires at least two repeats, while the repeat order and connection in sequence are less important^82^. The data suggest that each Tau repeat can be regarded as a single interaction motif. The number of Tau repeats controls the valency of interactions that drive LLPS, similar to multiple SH3 domains in the protein LAT^36^. Cellular bodies thus not only contain splicing factors^83^ and pre-mRNA splicing can occur in subnuclear speckles^84^, but alternative splicing might regulate the physiochemical properties of liquid-like compartments through controlling the multivalency of intermolecular interactions.

The wild-type Tau sequence contains two cysteine residues, C291 and C322^85^. C291 and C322 are located in the Tau repeats R2 and R3, respectively. Oxidation of the two cysteine residues has a strong influence on the ability of Tau to form amyloid fibrils^46^. Importantly, the oxidation state of the cysteines and the number of Tau repeats is strongly coupled, because C291 is only present in 4R-isoforms of Tau. Indeed, while oxidation promotes fibrillization of 3R-Tau through intermolecular disulfide bond formation, cysteine oxidation in 4R-Tau delays aggregation, because of the formation of intramolecular S–S bridges between C291 and C322^46^. Thus, at first sight the finding that K19 has a much lower propensity for LLPS when compared to K18 (Fig. 6) seems to be in disagreement with widely published aggregation data, which consistently reported more efficient fibrillization of K19/3R-Tau when compared to K18/4R-Tau^23, 46, 48^. However, this conflict is resolved when the oxidation/reduction state in the experiments is considered. In a reducing environment both 3R-Tau and 4R-Tau have a very low propensity to form amyloid fibrils and both require co-factors (e.g., heparin) to aggregate into amyloid fibrils. Because in living cells a highly reducing environment is found due to the presence of glutathione, we performed all experiments for K18-LLPS in reducing conditions. Nevertheless, it will be interesting to investigate in future studies how oxidation of Tau’s native cysteine residues influences its ability to undergo LLPS. In addition, studies of Tau and other proteins are required to investigate a possible connection between LLPS and cell-to-cell transmission of Tau aggregates^86^.

## Methods

### Protein preparation

K18 comprises all four repeats of the largest Tau isoform (2N4R; residues Q^244^-E^372^ plus initial M^243^), K19 is similar but lacks the second repeat, corresponding to fetal Tau (residues M^243^ + Q^244^-K^274^, V^337^-E^372^, without R2 = V^275^-S^305^). K25 comprises the projection domain of 0N4R Tau up to L^185^ (according to 0N4R notation; corresponding to L243 in 2N4R Tau). All constructs were expressed overnight at 20 °C in *Escherichia coli* strain BL21(DE3)^13^ from a pNG2 vector (a derivative of pET-3a, Merck-Novagen, Darmstadt) in 1–10 L LB for unlabeled and M9 minimal medium for labeled protein, induction with 0.5 mM IPTG at O.D. 600. The expressed proteins were purified from bacterial extracts by making use of the heat stability of the protein and by FPLC SP-Sepharose chromatography (Amersham Biosciences). The cell pellets were resuspended in boiling extraction buffer (50 mM MES, 500 mM NaCl, 1 mM MgCl_2_, 1 mM ethylene glycol tetraacetic acid (EGTA), 5 mM dithiothreitol (DTT), pH 6.8) complemented with a protease inhibitor mixture. The cells were disrupted with a French pressure cell and subsequently boiled for 20 min. The soluble extract was isolated by centrifugation, the supernatant was dialyzed against two changes of cation exchange chromatography buffer A (20 mM MES, 50 mM NaCl, 1 mM EGTA, 1 mM MgCl_2_, 2 mM DTT, 0.1 mM phenylmethylsulfonyl fluoride, pH 6.8) and loaded on an FPLC SP-Sepharose column. The proteins were eluted by a linear gradient of cation exchange chromatography buffer B (20 mM MES, 1 M NaCl, 1 mM EGTA, 1 mM MgCl_2_, 2 mM DTT, 0.1 mM phenylmethylsulfonyl fluoride, pH 6.8). Protein breakdown products were separated in a second chromatography step by using a Superdex G200 column (GE Healthcare) with the separation buffer (137 mM NaCl, 3 mM KCl, 10 mM Na_2_HPO_4_, 2 mM KH_2_PO_4_, pH 7.4, 1 mM DTT). To label K18 with ^15^N and ^13^C stable isotopes, the *E. coli* culture expressing K18 protein was grown in a M9 minimal medium with ^15^NH_4_Cl (1 g l^−1^) and ^13^C glucose (4 g l^−1^). Final samples contained 0.9–1.3 mM ^15^N-labeled or ^15^N/^13^C-labeled protein in 95% H_2_O, 5% D_2_O, 50 mM phosphate buffer, pH 6.8, with 1 mM DTT. The two native cysteine residues of Tau, C291 and C322, could therefore not undergo oxidation during the experiments.

To label K18 at the two native cysteines with the nitroxide spin label MTSL (Toronto Research Chemicals, ON, Canada), DTT was removed from the buffer by slide-A-lyzer mini dialysis device (MWCO 3500, Thermo Fisher Scientific), followed by equilibration in 50 mM sodium phosphate, pH 8.8. Free sulfhydryl groups were reacted with a five-fold molar excess of MTSL solubilized in ethyl acetate, at 4 °C for 12 h. Unreacted spin label was removed by dialysis (as mentioned above). Spin-labeled proteins were concentrated using Amicon Ultra-15 centrifugal filters (molecular weight cutoff of 3000; Millipore, Cork, Ireland).

### Phosphorylation of K18 by MARK2

With the aim of achieving a high degree of phosphorylation, K18 was incubated at 25 °C with MARK2cat-T208E at a Tau:MARK2 ratio of 100:1 for 16 h. The buffer contained 25 mM 2-[4-(2-hydroxyethyl)piperazin-1-yl]ethanesulfonic acid (pH 8.0), 100 mM NaCl, 5 mM MgCl_2_, 2 mM EGTA, 1 mM DTT, 1 mM benzamidine, 0.5 mM phenylmethanesulfonyl fluoride, and 1 mM ATP. After phosphorylation, Tau samples were buffer exchanged with 50 mM NaH_2_PO_4_/Na_2_HPO_4_ (pH 6.8) and 10% (v/v) D_2_O. The sites of phosphorylation, as well as the degree of phosphorylation at each specific site was quantified according to previously published procedures^79^ using 2D ^1^H-^15^N HSQC spectra of the phosphorylated K18 sample and a non-phosphorylated K18 sample, both recorded at 5 °C on a Bruker 700 MHz NMR spectrometer.

### Turbidity measurements

Turbidity of protein samples was estimated from the optical density at 350 nm, recorded on an Infinite M1000Pro plate reader (Tecan) using flat bottom 96-well plates (Greiner). For temperature-dependent measurements, heating or cooling was performed at a rate of 1 °C min^−1^. Protein concentrations and buffer conditions are specified in the Fig. legends (Figs. 2a,b, 3a, 6b,c).

### Far-UV CD spectroscopy

Far-UV CD measurements were performed on a Jasco-815 spectropolarimeter equipped with Peltier cell temperature controller (±0.2 °C) using 0.1 cm path length cuvette. Typical protein concentrations were 0.1–0.2 mg ml^−1^ in 25 mM sodium phosphate (pH 8.8). CD spectra were recorded from 260 to 190 nm with a scanning speed of 20 nm min^−1^ and 10 accumulations per scan. Spectra were baseline corrected using buffer. All measurements were recorded in duplicate.

### ThT fluorescence

Protein aggregation was monitored by a standard assay for probing the presence of amyloid fibrils^48, 82^, which is based on the binding of ThT to cross-β-structure. To this end, 100 μM of K18 was incubated at 37 °C in presence or absence of heparin and with various NaCl concentrations (as mentioned in figures). According to well-established procedures for ThT fluorescence measurements^48, 82^, 1 μM of protein was mixed with 10 μM of freshly prepared ThT solution. Spectra were measured between 460 and 600 nm on Varian Cary Eclipse fluorescence spectrophotometer (Agilent Technologies) using an excitation wavelength of 442 nm.

### DIC and confocal microscopy

Droplet formation of protein samples was monitored by DIC microscopy. 15 μl of sample were loaded onto glass coverslips and DIC images were acquired on a DM6000B (Leica) microscope with a 63×-objective (water immersion). Alexa-488-labeled protein was prepared using Alexa Fluor 488 microscale protein labeling kit (Thermo Fisher Scientific). To confirm the presence of protein in the droplets, Alexa-488-labeled protein was mixed with unlabeled protein and the images were acquired on a DM6000B (Leica) microscope with a 63×-objective (water immersion) using a 488 nm argon laser line. Images were processed using ImageJ.

### Transmission electron microscopy

For electron microscopy, the aggregated K18 sample was bound to a glow discharged carbon foil covered grid. After staining with 1% uranyl acetate, samples were evaluated at room temperature with a CM 120 transmission electron microscope (FEI, Eindhoven, the Netherlands) using a TemCam F416 CMOS camera (TVIPS, Gauting, Germany).

### NMR spectroscopy

To study the interaction of K18 with heparin, two-dimensional ^1^H-^15^N HSQC spectra were recorded on a Bruker 700 MHz spectrometer, equipped with a triple resonance cryogenic probe, at 5 °C either for the protein (50 μM K18 in 50 mM sodium phosphate, pH 8.8, 0.5 mM TCEP, 0.1 mM DSS, and 10% D_2_O) alone or using a 4:1 K18:heparin molar ratio. Spectra were acquired with 1024 and 256 complex data points in the direct and indirect dimension, respectively, and were processed using Bruker Topsin 2.1. ^1^H chemical shifts were calibrated relative to DSS and ^15^N chemical shifts were referenced indirectly.

Paramagnetic relaxation enhancement (PRE) effects of NMR signals were extracted from the peak intensity ratios between 2D ^1^H-^13^C-HSQC NMR spectra acquired for MTSL-labeled K18 (100 μM K18 in sodium phosphate, pH 8.8; paramagnetic state) and after addition of 1 mM DTT (heated to 42 °C for 30 min before measurement) to the same sample. Addition of DTT cleaves the MTSL tag from the cysteine residue such that the spin label is no longer attached to the protein and the protein is in the diamagnetic state. We previously showed that oxidation of the MTSL tag by ascorbic acid gives very similar results in the case of Tau^19^. For PRE profiles, the peak intensity of every residue in the ^1^H-^13^C-HSQC, which was recorded for the sample with the MTSL-tag attached to the two native cysteine residues (C291 and C322), was divided by the peak intensity in the ^1^H-^13^C-HSQC with the cleaved tags (PRE = *I*_para_/*I*_dia_). Measurements were performed at 5 °C where K18 is present as dispersed monomeric protein, and at 37 °C where DIC microscopy showed that the K18 solution has undergone LLPS. Resonance assignments of K18 were previously determined^55^ and used for the identification of the Cα-Hα cross-peaks of the four threonine residues in K18.

### Data availability

All relevant data are available from the corresponding author upon reasonable request.
